# Anatomic Evaluation of the Quadriceps Tendon in Cadaveric Specimens: Application for Anterior Cruciate Ligament Reconstruction Graft Choice

**DOI:** 10.51894/001c.7961

**Published:** 2019-07-01

**Authors:** Nathan Krebs, Amjad Yaish, Nicholas O’Neill

**Affiliations:** 1 Orthopedic Surgery Residency - Graduate Medical Education McLaren Macomb Regional Medical Center; 2 Orthopedic Surgery McLaren Macomb Regional Medical Center

**Keywords:** cadaveric study, ligament autograft, anterior cruciate ligament, quadriceps tendon

## Abstract

**Purpose:**

The quadriceps tendon (QT) is an autograft option for primary and revision anterior cruciate ligament (ACL) reconstruction. Techniques for predicting the appropriate graft size are limited. The purpose of this study was to evaluate the morphologic features of the QT in cadaveric specimens and compare the findings to recent MRI studies.

**Materials and Methods:**

Macroscopic dissections were performed on 10 cadaveric knees. Using the distal myotendinous junction of the rectus femoris and superior pole of the patella as anatomic landmarks, the QT was isolated. Tendon length, width, and thickness were recorded at 10 millimeter (mm) increments. A central 80 mm x 10 mm graft was harvested, after which the graft was measured in an identical fashion. Specimen anthropometric data was collected. Subgroup analysis and linear regression were then performed using Microsoft Excel 2011 Office Analysis ToolPak.

**Results:**

The mean QT length was 83.3 +/- 14.4 mm, ranging from 63 to 108 mm. The mean percentage of remaining QT volume following graft harvesting was 63.3%. QT length showed significant correlation with patient height (correlation coefficient: 0.719, p = 0.027). QT thickness remained relatively constant, while the width is greatest at its patellar insertion and gradually decreases proximally towards the myotendinous junction.

**Conclusion:**

The QT has the anatomical features to produce a robust autograft for ACL reconstruction. During preoperative evaluation of graft size and quality, patient height should be considered as it is strongly correlated with the length of the potential graft. Our findings support the use of MRI as a way to preoperatively assess the QT as an autograft when performing an ACL reconstruction.

## INTRODUCTION

There has been a recent resurgence of interest in the use of the quadriceps tendon (QT) as a potential graft choice in primary anterior cruciate ligament (ACL) reconstructions.^1–3^ The most utilized autograft choices have historically been bone-patella tendon-bone (BPTB) and the hamstrings, which have both proven to be reliable in terms of graft quality and clinical outcomes.^4^ Although the QT has provided a favorable graft option in certain settings,^5^ the indications for its use may begin to expand.^6^ Although the magnetic resonance imaging (MRI) assessment of QT characteristics has recently been shown to correlate with intraoperative autograft findings,^7^ there is still limited data on the gross anatomic features of the tendon.

The purpose of our study was to analyze the morphology of the QT and correlate the measurements to comparable studies done using MRI. Our hypothesis was that the morphological features of the QT in cadaveric specimens would be concordant with data produced from QT assessment done by MRI studies.

## MATERIALS and METHODS

**Figure 1:**
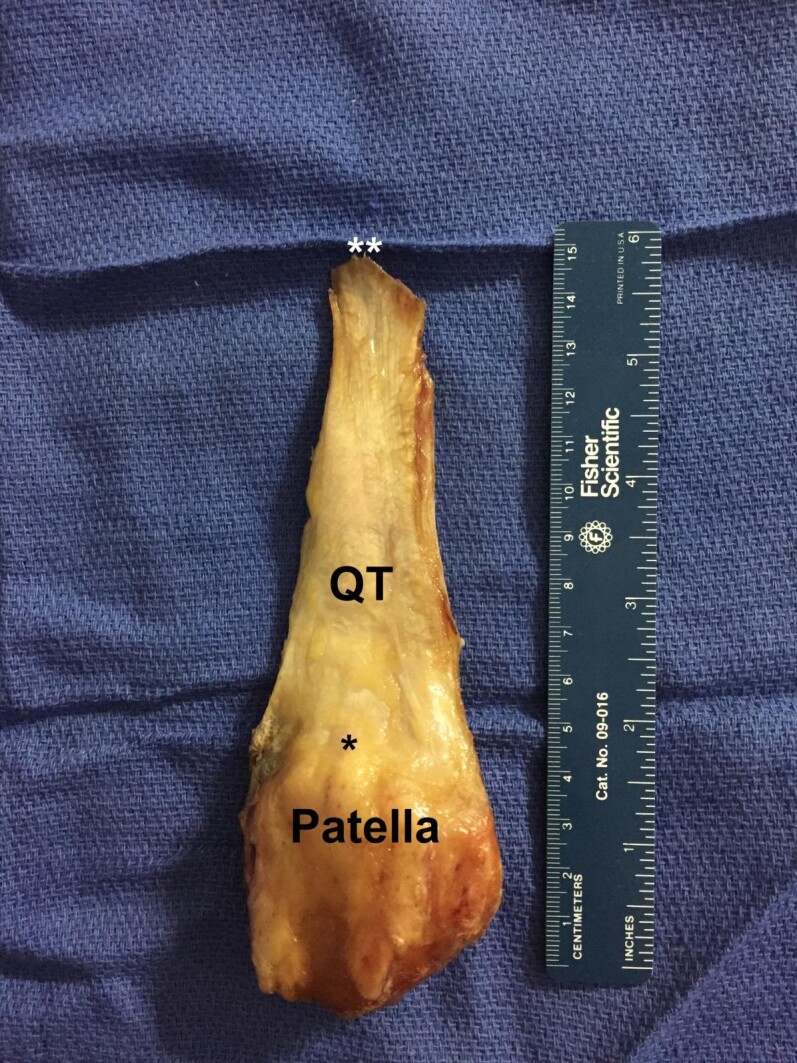
Dissected quadriceps tendon (QT). Its total length is defined by the distance between the myotendinous junction of the rectus femoris (two white asterisks) and its patellar insertion (black asterisk)

Macroscopic dissections were performed on ten adult, fresh-frozen cadaveric lower extremities (six males and four females). Known anthropometric data including age, height, weight, and body mass index (BMI) were recorded. The superficial skin and soft tissues were removed to expose the quadriceps tendon, patella, and patellar tendons. The fibers of the medial and lateral portions of the quadriceps muscles were detached to isolate the quadriceps tendon. Proximally, the QT tendon was released at the musculotendinous junction of the quadriceps muscle. Distally, the quadricep tendon’s insertion on the patella was maintained, after which the patellar ligament and medial and lateral patellar retinacula were removed [Figure 1].

**Figure 2:**
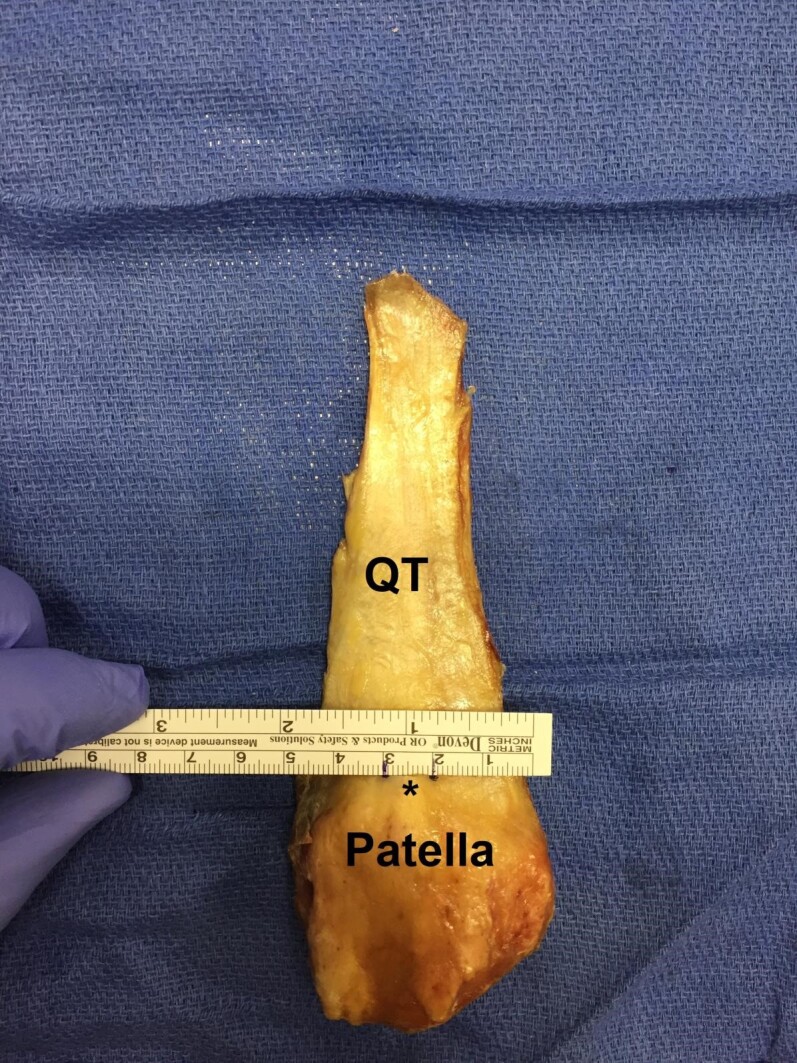
A 10 mm central portion of the quadriceps tendon (QT) was identified by measuring the width of the tendon at the level of its patellar insertion and dividing that measurement in half (black asterisk)

Using a standard operative ruler, the length of each tendon was measured and recorded to the nearest millimeter (mm). The most distal portion of the rectus femoris was used to objectively delineate the most proximal portion of the quadriceps tendon. The width and thickness were measured at nine predetermined locations along the tendon, beginning at its insertion on the patella and moving proximally by 10 mm increments, ending at a level 80 mm proximal to its insertion. The central portion of the tendon was identified by measuring the width of the tendon at its patellar insertion [Figure 2].

**Figure 3:**
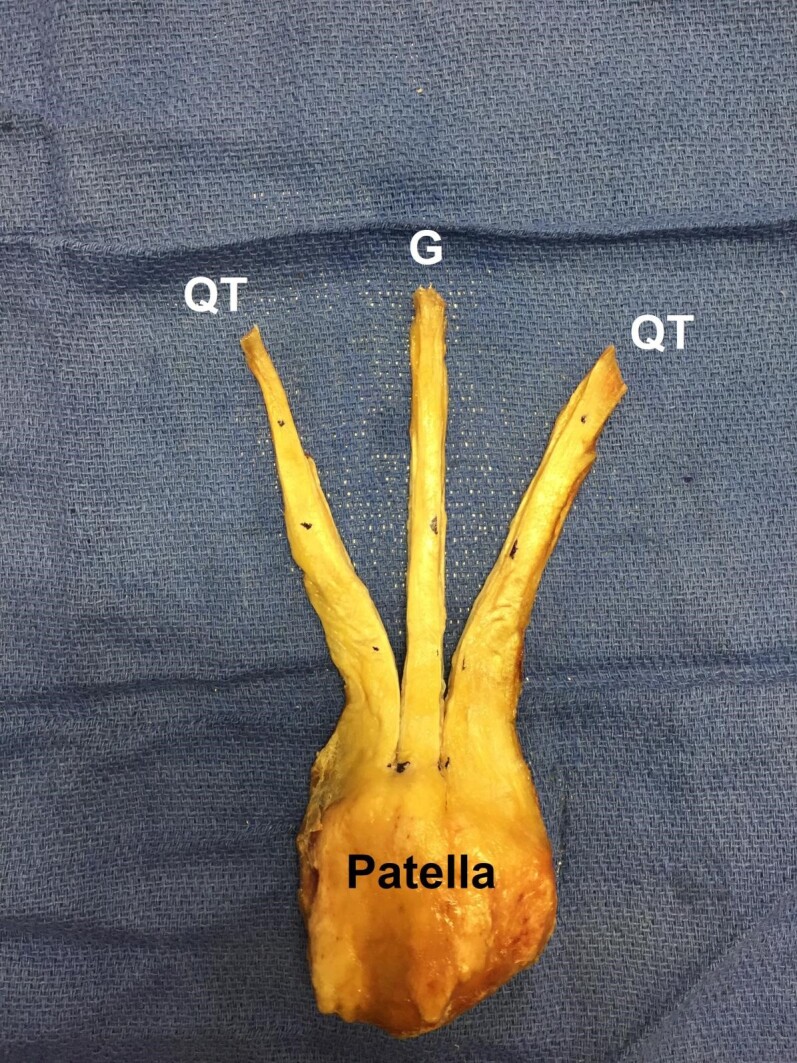
A 10 mm wide graft (G) was dissected longitudinally along the quadriceps tendon (QT) length.

Using the same operative ruler, 10 mm of the central tendinous insertion site was measured and cut proximally along its length [Figure 3].

**Figure 4:**
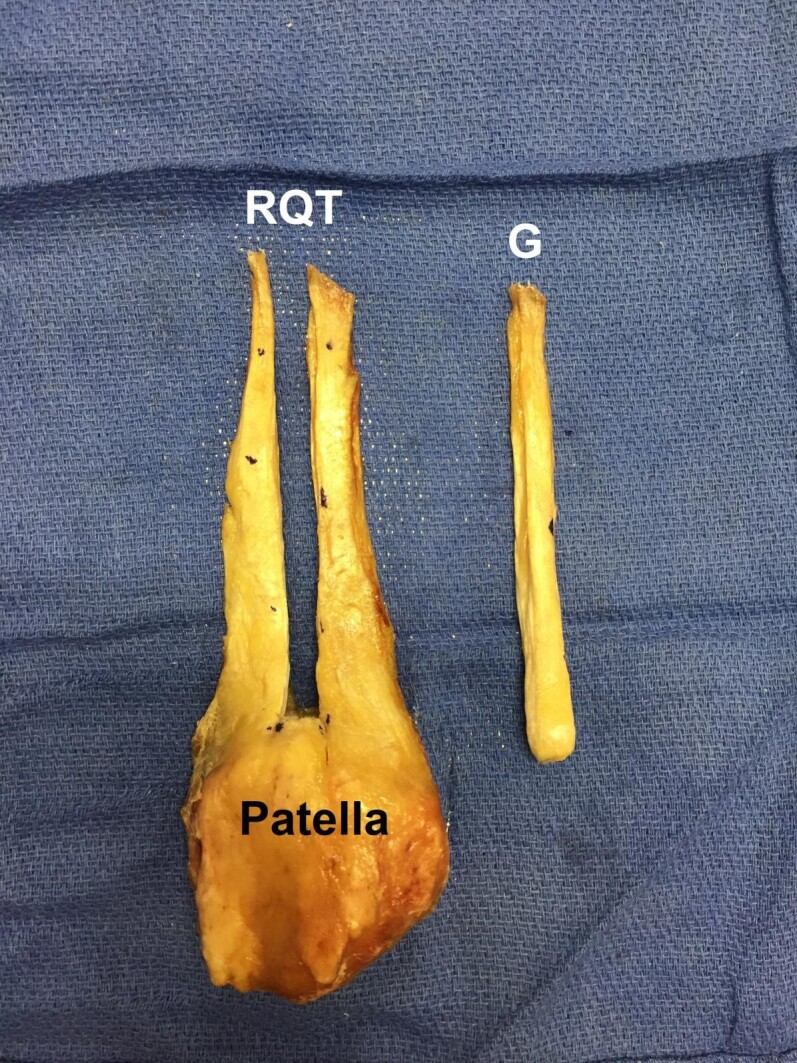
Once the graft (G) harvest was complete, final measurements of the remaining quadriceps tendon (RQT) volume were recorded.

An 80 mm x 10 mm graft was then harvested [Figure 4].

Once the graft was harvested and removed, the remaining percentage of QT volume was measured and recorded. All measurements of each specimen were taken in the same fashion by the second author. The first author then analyzed the collected data using Microsoft Excel 2011 Office Analysis ToolPak and performed subgroup and linear regression analyses.

## RESULTS

The mean length of the QT segments in this study was 83.3 +/- 14.4 mm (measurements ranged from 63 to 108 mm). The mean percentage of tendon volume remaining following removal of an 80 mm x 10mm graft from the QT was 63.3%. This value was done by calculating the volume of the native QT and the volume of the QT graft, then subtracting the value of QT graft from the native QT volume. The correlation coefficients of the tendon size relative to each anthropometric value can be seen in Table 1.

**Table 1.** Correlation Coefficients between Tendon Size and Anthropometric Data

	**Correlation value (Pearson), ρ**	**Level of significance**
Age (years)	-0.497	p = 0.140
Height (inches)	0.719	p = 0.027
Weight (lbs)	0.596	p = 0.084
Body mass index (BMI)	0.042	p = 0.987

Correlation coefficient for height, P<.05

The height of the cadaver had the highest correlation to quadriceps tendon length (correlation coefficient: 0.719, p = 0.027) [Graph 1].

**Graph Graph 1.**
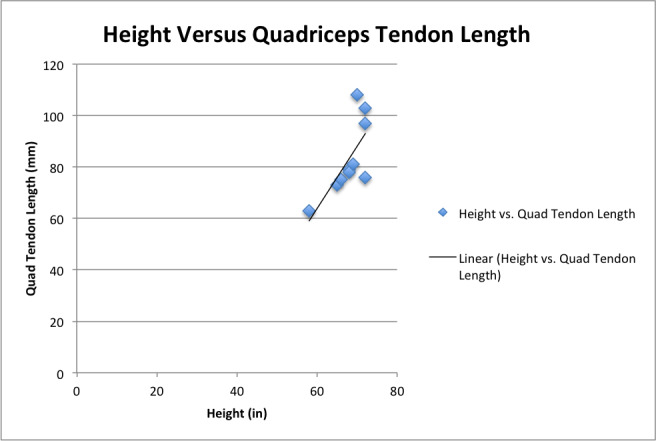
Linear Relationship established between Cadaver Height and Quadriceps Tendon Length

The thickness of the QT was relatively constant, although it does thin proximally near its origin. The width is greatest at the insertion of the patella and decreases as it goes proximally to the myotendinous junction [Table 2].

**Table:** Table 2 Change in Quadriceps Tendon Width relative to Distance from Patellar Insertion

	**QT^b^ Width (mm)**		**Percentage of MW^d^ (%)**
**Distance from PI^a^ (mm)**	**Mean +/- SD^c^**	**Range**	
Insertion	44.8 +-/ 2.8	43 – 49	100.0
10 mm	41.4 +-/ 2.9	39 – 46	92.4
20 mm	35.0 +-/ 2.9	33 – 41	78.1
30 mm	29.2 +-/ 3.8	26 – 35	65.1
40 mm	25.4 +-/ 4.3	22 – 33	56.6
50 mm	21.6 +-/ 4.5	18 – 30	48.2
60 mm	18.4 +-/ 3.8	16 – 27	41.1
70 mm	16.1 +-/ 2.6	15 – 21	35.9
80 mm	14.2 +-/ 2.3	12 – 19	31.6

^a^PI; patellar insertion site

^b^QT; quadriceps tendon ^c^SD;

standard deviation

^d^MW; Maximum width of quadriceps tendon measured at level of patellar insertion

• aSD, standard deviation

• b Percentage when each location is compared with the width at insertion on to patella

Subgroup analyses were performed by separating the cadavers into two groups based on their overall height. Group 1 (n=2) included cadavers that were 58 to 65 inches tall and had a mean QT length of 68.0 mm +/- 7.1 mm. Group 2 (n=8) included cadavers that were 66 to 72 inches tall and had a mean QT length of 87.1 mm +/- 13.3 mm. Although approximately 20 mm separated the two groups, these findings were not statistically significant. [Table 3].

**Table:** Table 3 Subgroup Analysis of Quadriceps Tendon Length versus Cadaver Specimen Height

**Group (height)**	**Specimen number (n)**	**QT length (mm)**	
		**Mean +/- SD^a^**	**Range**
1 (58 – 65 inches)	2	68.0 +-/ 7.1	63 – 73
2 (66 – 72 inches)	8	87.1 +-/ 13.3	75 – 108
All	10	83.3 +-/ 14.4	63 – 108

^a^SD; standard deviation

• Quad Tendon Length (mm)

## DISCUSSION

Although BPTB and hamstrings autografts have historically served as the primary graft options for ACL reconstructions, the favorable clinical outcomes observed with the use of QT autograft have recently received considerable attention.^8–12^ With the advent of modern surgical techniques for safely harvesting the QT graft,^13^ contemporary data has demonstrated various clinical applications for its use in both primary and revision ACL reconstructions.^3,14–16^

However, accurately predicting the size and quality of any ACL autograft option during surgical planning is difficult. Both cadaveric and clinical imaging studies of ligamentous structures around the knee joint have done well to show high correlations between MRI measurements and anatomic findings when analyzing these structures in laboratory dissections.^17,18^ As it relates to orthopedic anatomy in general, these types of studies have proven to be fundamental in defining the functional importance of complex ligamentous structures in other areas of the body as well.^19^ This data shows that, in addition to anthropometric patient information, preoperative MRI autograft measurements are reliable and can assist the surgeon in choosing the appropriate graft relative to the patient’s needs.

The QT has the anatomic features to produce a robust graft, which appears to be reproducible and is comparable to the proven bone-patella tendon-bone graft in regards to volume. As demonstrated in previous biomechanical cadaveric studies, the cross-sectional area of the quadriceps tendon graft may be nearly twice that of the

BTPB, which contributes to the QT graft’s higher stiffness and ultimate load to failure.^20^

Patient height is also an important variable when considering the use of the QT as it is strongly correlated with length of the potential graft. Zakko *et al.* showed similar correlations between patient anthropometric data and quadriceps tendon size, suggesting that this information can be used to reliably predict the appropriate graft size prior to surgery.^18^ These findings coincide with contemporary data on using patient height as an indicator of patellar ligament graft length, a widely used autograft in ACL reconstruction surgery.^21,22^

In this study, we found that the volume of the QT graft and the percentage of the remaining tendon are greater than what has been previously reported of the bonepatella tendon-bone graft in similar anatomic studies using 3D MRI techniques.^23^ This is advantageous as the strength of the graft and viability of the remaining in vivo tendon has been proven to be correlated to the cross-sectional volume.^24^

Kanakamedala *et al.* performed a systematic review of 20 level III and level IV studies analyzing the clinical outcomes of full and partial thickness quadriceps tendon autografts in primary ACL reconstructions.^25^ They found that QT autografts appeared to have successful outcomes and low failure rates, regardless of graft thickness. Lee *et al.* compared bone-QT graft versus double-bundle hamstrings graft and found that the bone-QT cohort had similar knee stability and better flexor muscle strength recovery when compared to the double-bundle hamstrings cohort.^26^ Emerging data suggests that the clinical outcomes with the use of QT autografts are comparable, and arguably superior, to those seen with the use of other autograft options.^2^

**Figure 5:**
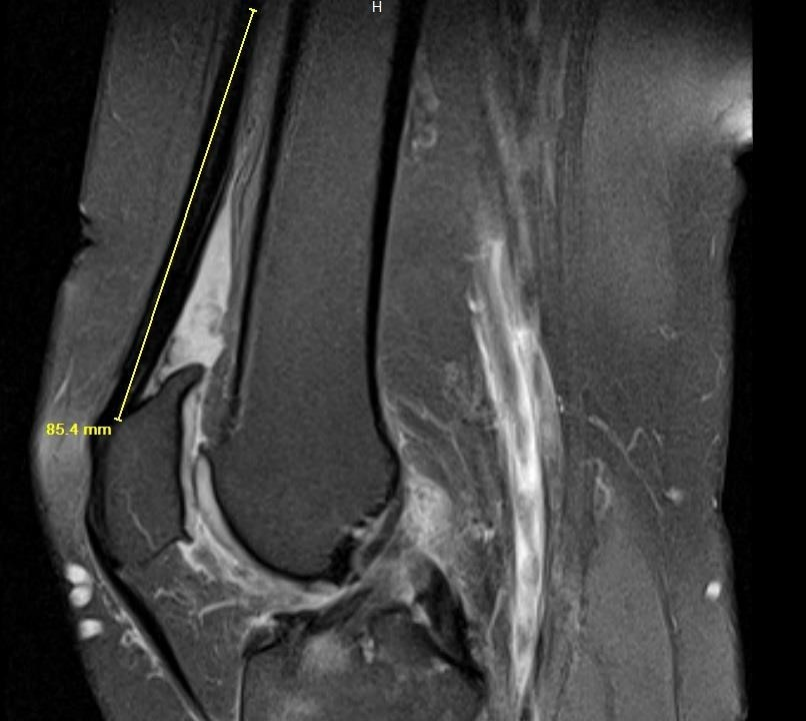
Midsagittal MRI image of the knee using the superior pole of the patella (distal endpoint) and musculotendinous junction of the rectus femoris muscle (proximal endpoint) as anatomic landmarks to measure the QT preoperatively.

Slone et al. describes a minimally invasive technique of quadriceps tendon harvesting that involves making a longitudinal incision from the middle portion of the superior pole of the patella to 2 cm proximal, centered over the tendon with the knee in 90 degrees of flexion.^3^ The tendon is exposed and the central 10 mm of tendon is harvested from the proximal pole of patella to 7 cm proximal with careful attention to not violate the capsule. The most desirable graft length is 7 cm, with the absolute minimal length requirement of 6.5 cm.^3^ It has been shown that 90% of patients 5 feet 6 inches or taller had a QT length of at least 70 mm in the study by in the study by Xerogeanes et al.^23^ In patients shorter than this, preoperative MRI measurements are recommended to evaluate potential graft length. This may be done by measuring the midsagittal view of the tendon from the superior pole of the patella to the musculotendinous junction of the rectus femoris [Figure 5].^3,23^

## CONCLUSIONS

As the number of primary ACL reconstructions, as well as revisions, have been increasing in the United States, it is critical for the orthopedic surgeon to be cognizant of the numerous types of graft options. Additionally, it is important that surgeons consider the unique aspects of each their patients when considering which autograft to harvest. The findings in this study support the use of MRI as a means of preoperatively assessing the QT as an autograft option when performing an ACL reconstruction.

### Conflict of Interest

The authors declare no conflict of interest.
